# Effects on the Upper Airway Morphology with Intravenous Addition of Ketamine after Dexmedetomidine Administration in Normal Children

**DOI:** 10.3390/jcm9113723

**Published:** 2020-11-20

**Authors:** Goutham Mylavarapu, Robert J. Fleck, Michale S. Ok, Lili Ding, Ali Kandil, Raouf S. Amin, Bobby Das, Mohamed Mahmoud

**Affiliations:** 1 Cincinnati Children’s Hospital Medical Center, Division of Pulmonary Medicine, Cincinnati, OH 45229, USA; Raouf.Amin@cchmc.org; 2 Cincinnati Children’s Hospital Medical Center, Department of Radiology, Cincinnati, OH 45229, USA; Robert.Fleck@cchmc.org; 3 Cincinnati Children’s Hospital Medical Center, Department of Anesthesiology, Cincinnati, OH 45229, USA; Michale.Ok@cchmc.org (M.S.O.); Ali.Kandil@cchmc.org (A.K.); Bobby.Das@cchmc.org (B.D.); Mohamed.Mahmoud@cchmc.org (M.M.); 4 Cincinnati Children’s Hospital Medical Center, Division of Biostatistics and Epidemiology, Cincinnati, OH 45229, USA; Lili.Ding@cchmc.org

**Keywords:** dexmedetomidine, ketamine, intravenous, sedation, MRI, upper airway, pediatric, anesthesia

## Abstract

General anesthesia decreases the tone of upper airway muscles in a dose-dependent fashion, potentially narrowing the pharyngeal airway. We examined the effects of adding ketamine on the airway configuration after dexmedetomidine administration in spontaneously breathing children with normal airways. 25 children presenting for Magnetic Resonance Imaging (MRI) of the brain/spine under general anesthesia were prospectively recruited in the study. Patients were anesthetized with dexmedetomidine bolus (2 mcg over 10 min) followed by dexmedetomidine infusion (2 mcg·kg${}^{-1}$·h) and ketamine and permitted to breathe spontaneously via the native airway. MR-CINE images of the upper airway were obtained with dexmedetomidine infusion alone (baseline) and 5, 10, and 15 min after administering ketamine bolus (2 mg·kg${}^{-1}$) in two anatomical axial planes at the nasopharynx and the retroglossal upper airway. Airway lumen is segmented with a semi-automatic image processing approach using a region-growing algorithm. Outcome measures of cross-sectional area, transverse and anterior-posterior diameters of the airway in axial planes at the level of the epiglottis in the retroglossal airway, and in the superior nasopharynx were evaluated for changes in airway size with sedation. Airway dimensions corresponding to the maximum, mean, and minimum sizes during a respiratory cycle were obtained to compare the temporal changes in the airway size. The dose-response of adding ketamine to dexmedetomidine alone condition on airway dimensions were examined using mixed-effects of covariance models. 22/25 patients based on inclusion/exclusion criteria were included in the final analysis. The changes in airway measures with the addition of ketamine, when compared to the baseline of dexmedetomidine alone, were statistically insignificant. The modest changes in airway dimensions are clinically less impactful and within the accuracy of the semi-automatic airway segmentation approach. The effect sizes were small for most airway measures. The duration of ketamine seems to not affect the airway size. In conclusion, adding ketamine to dexmedetomidine did not significantly reduce upper airway configuration when compared to dexmedetomidine alone.

## 1. Introduction

The unique sedative properties of dexmedetomidine (DEX) have renewed interest in the study and use of alpha 2-adrenoceptor agonists as sedatives and/or adjuncts to anesthesia in a variety of pediatric procedures [1,2,3]. Dexmedetomidine’s pharmacological profile, specifically its sympatholytic and anesthetic-sparing effects coupled with the preservation of respiratory drive, makes it a very useful sedative in the pediatric population. Dexmedetomidine induced sedation mimics the physiologic changes seen during non-rapid-eye-movement sleep through its action on alpha 2-adrenergic receptors in the locus ceruleus. Besides, there is increasing evidence supporting its protective effects against ischemic and hypoxic injury [4,5,6,7].

Although dexmedetomidine provides effective sedation for non-invasive procedures, it has been largely unsuccessful in providing adequate analgesia for painful procedures when used alone [8,9,10]. Administering high doses of dexmedetomidine to provide adequate sedation for procedures may lead to significant hemodynamic instability, specifically bradycardia, and swings in mean arterial pressure. Concurrent administration of ketamine with dexmedetomidine appears to mitigate these dexmedetomidine-induced hemodynamic changes [11]. Combining ketamine with dexmedetomidine has been studied in adults and children for drug-induced sleep endoscopy (DISE) [12], extracorporeal shock wave lithotripsy [13], lumbar puncture [14], bone marrow biopsy, burn dressing changes [15,16], chest tube insertion, and femoral cut-down for tunneled central venous catheter placement [17].

The effect of dexmedetomidine on upper airway collapsibility in normal and obstructive sleep apnea patients has been studied [18,19,20], however, there is no literature describing the effects of combining dexmedetomidine and ketamine on upper airway morphology in children. The primary objectives of the study are to examine the effects of adding ketamine after dexmedetomidine administration on various measures of airway size and configuration (diameter, cross-sectional area) derived from Magnetic Resonance images (MRI) of the native airways of spontaneously breathing children. We hypothesized that adding ketamine to dexmedetomidine would not significantly reduce airway caliber or morphology when compared with dexmedetomidine alone.

## 2. Materials and Methods

This study was registered at www.clinicaltrials.gov (NCT02652507). The Institutional Review Board of Cincinnati Children’s Hospital Medical Center reviewed and approved this study. Written, informed consent was obtained from the subject’s parents. Children ages 1 to 18 years (inclusive) presenting for elective MR examination under general anesthesia were recruited. This study is not a randomized controlled trial so no randomization will be used in this study. All subjects were required to have planned MR imaging of the brain or brain/spine under general anesthesia. Exclusion criteria included severe comorbidities (American Society of Anesthesiologists (ASA) Physical Status > 2), history of obstructive sleep apnea or snoring, allergy to dexmedetomidine or ketamine, neurological disorders, cerebral palsy or demyelinating disease which may affect airway muscle tone, and malignant hyperthermia.

### 2.1. Patient Sedation

No pre-medications were administered. Anesthesia was induced with sevoflurane with or without nitrous oxide. An IV catheter was inserted, and inhalational agents (e.g., sevoflurane and nitrous oxide) were discontinued. Next, all patients received an IV loading dose of dexmedetomidine (2 μg·kg${}^{-1}$) over 10 min followed by dexmedetomidine infusion at 2 μg·kg${}^{-1}\cdot$h. After confirmation of adequate sedation, patients were positioned supine with the cervical spine in the neutral position on the MRI table. All patients continued to breathe spontaneously and all subjects had an end-tidal sevoflurane concentration of less than 0.1% before transfer to the MRI scanner. No attempt was made to open or close the mouth. Subjects were then transferred into the MRI scanner, where they spontaneously breathed supplemental oxygen (2 L·min${}^{-1}$) via nasal cannula. No artificial airway (e.g., oral airway or nasal trumpet) or positioning aid (e.g., shoulder roll) was used during MRI. Upon completion of the image acquisition, subjects were transferred to a recovery room. Monitoring included continuous electrocardiography and pulse oximetry, as well as intermittent automated blood pressure measurements every 3 min. End-tidal CO${}_{2}$ was monitored.

Baseline airway images were obtained during dexmedetomidine infusion (2 mcg·kg${}^{-1}$·h). If the subject moved, a bolus of 0.5 mcg·kg${}^{-1}$·h over 1 min was administered and the dexmedetomidine infusion rate was increased to 2.5 mcg·kg${}^{-1}$·h. If the subject moved a second time, the research study was terminated, and additional anesthesia (propofol infusion) was provided. After initial airway images were obtained, a bolus dose of ketamine 2 mg·kg${}^{-1}$ was administered. Airway images were then obtained at 5, 10, and 15 min after the ketamine administration. After the last set of research airway images were completed, the subject’s participation in the study was complete and the dexmedetomidine infusion was adjusted by the anesthesiologist to maintain an adequate depth of anesthesia for the remainder of the scheduled procedure. Dexmedetomidine bolus doses and infusion rates during the rest of the procedure adhered to the usual dosing for dexmedetomidine as a sole anesthetic agent for non-invasive procedural sedation in children [21]. Patients were discharged after meeting standard discharge criteria, including the level of consciousness (awake or easy arousal with verbal commands), core temperature 36 °C, ability to swallow (taking oral fluids), adequacy of muscle strength (strong and close to baseline movements of extremities and head), and status consistent with the patient’s preoperative baseline level of function.

Depth of sedation was assessed with the University of Michigan Sedation Scale (UMSS) [22]. UMSS is a simple, validated and reliable scale devised to facilitate rapid assessment and documentation of anesthetic depth of sedation in patients receiving sedative agents for diagnostic or therapeutic procedures. UMSS sedation scores were (0 awake/alert; 1 minimally sedated: tired/sleepy, an appropriate response to the verbal conversation and/or sounds; 2 moderately sedated: somnolent/sleeping, easily aroused with light tactile stimulation; 3 deeply sedated: deep sleep, arousable only with significant physical stimulation; 4 unarousable).

### 2.2. Imaging

Image acquisition for this study was performed on a 1.5 Tesla Philips Ingenia MRI scanner, such that the dynamic nature of airway caliber change over time could be captured [23]. The primary sequence was a fast gradient-echo sequence capable of obtaining two to three images per second in a prescribed plane and displayed to create a movie of the dynamic changes in the airway during breathing (CINE MRI). Sequence parameters: n at 1.5 T are FOV = 215 × 210 mm (frequency × phase), matrix (frequency × phase) = 128 × 82, TR/TE = 5.49/3.24 msec, flip angle = 15${}^{\circ }$, acceleration factor = 1.5, receiver bandwidth = 434 Hz, pixel, signal averages = 1) Subjects were imaged with dexmedetomidine alone (00 min, baseline) and 5, 10 and 15 min after the addition of ketamine. Approximately 72 consecutive images were obtained at the midpoint between the uvula and epiglottis, known as the retroglossal airway, and in an oblique axial plane where the velum and posterior pharyngeal wall are closest, termed the nasopharynx, during continuous breathing with a temporal resolution of 300 to 400 msec. These axial images were obtained at the baseline (00 min) condition, and 5, 10, and 15 min after administration of ketamine. This cine MRI allows us to quantify the temporal changes in morphology at the narrowest portion of the airway, in the nasopharyngeal (NP) airway; and in the region most affected by the dilator muscles, the retroglossal (RG) airways, respectively.

### 2.3. Image Analysis

**Figure 1:**
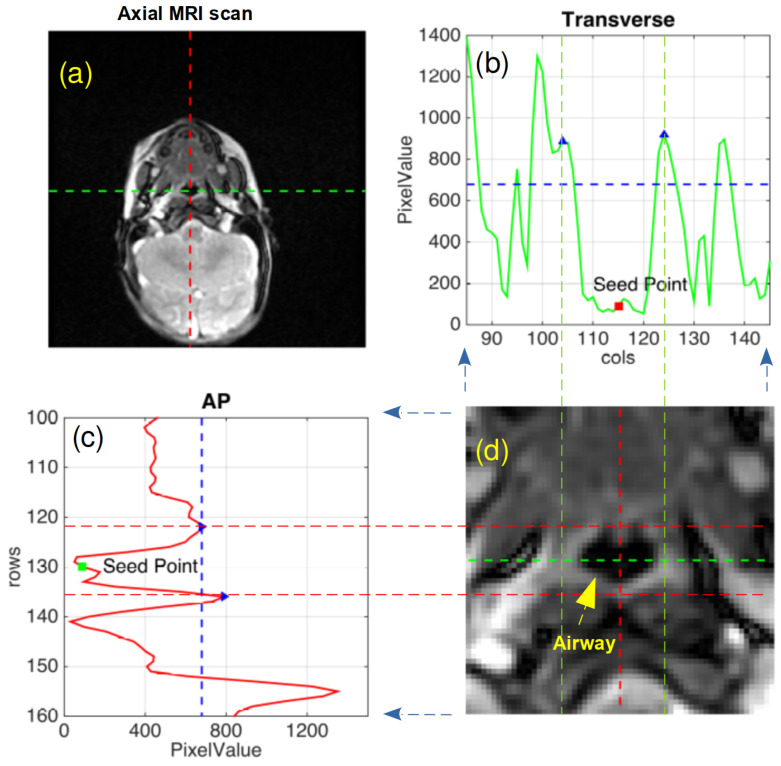
(**a**) A sample MR image at the nasopharyngeal plane of the upper airway, randomly selected from the 72 CINE MR images of continuous breathing in a study participant; Changes in pixel intensity values in the MR image along the (**b**) transverse (TX) and (**c**) anterior-posterior (AP) axes; (**d**) Cropped view of MR image shown in (**a**); The length and breadth of cropped image in (**d**) are aligned with rows and column axes. The blue triangles in (**b**) and (**c**) which are at the local peaks along TX and AP axes gives the extent of airway (red and green dashed lines) in (**d**).

The boundaries for the airway lumen in each of the 72 CINE MR images at both the NP and RG planes, were segmented at each of the four anesthetic conditions (DEX alone, and 5, 10, 15 min of ketamine administration) for every subject. The airway wall is segmented from the surrounding tissue in the axial MRI, based on a region-growing threshold algorithm [24] and using a semi-automated custom code written in MATLAB R2016 (Figure 1a–d). The segmentation process involves setting a user-defined seed on the first frame of the 72 images in a cine MRI sequence (Figure 1d). The seed is allowed to grow until it reaches the airway boundary with the surrounding tissue. The boundary of airway lumen is determined based on the pixel intensity changes in the transverse (TX) and anterior-posterior (AP) (Figure 1c,d) axes respectively, corresponding to the column and row axes in the MR image. The local peaks where the pixel values change significantly (black in the airway to gray outside the airway) is used to identify the rectangular limits of the airway boundary. All the low intensity value pixels within these rectangular limits are labeled in the MRI image as airway. Airway lumen is segmented in each of MRI using this automated workflow.

**Figure 2:**
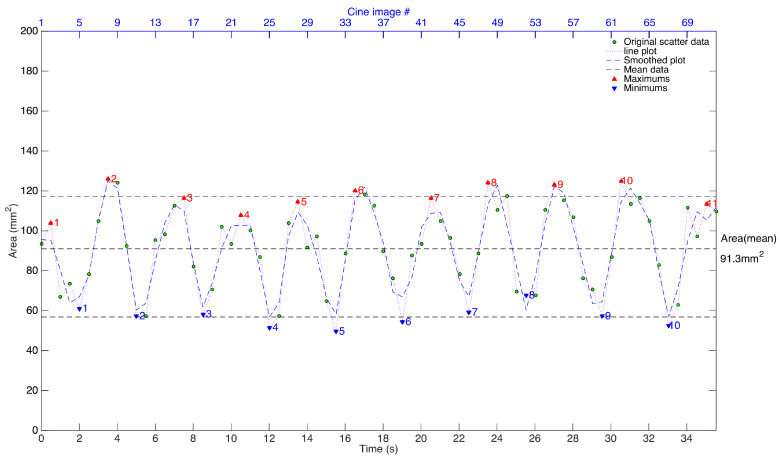
Changes in cross-sectional area of the airway across 72 consecutive images of cine MRI. The minimum and maximum airway dimensions are obtained by averaging all the minimums/valleys (down triangles) and maximums/peaks (up triangles) in the airway measure plotted as a function of time. The mean airway dimension is the average of all the 72 airway measures, representing the average size of the airway lumen.

The primary outcome measures of cross-sectional area (mm${}^{2}$) and the secondary outcomes are AP diameter (mm) and TX diameter (mm) of the pharyngeal airway measured at NP and RG planes following airway segmentation in each MR image; and arterial blood pressure, heart rate relative to baseline following DEX loading and infusion, and at 5, 10, and 15 min after adding ketamine to the anesthetic regimen. Temporal airway measures of cross-sectional area, AP and TX diameters during continuous breathing (72 frames of MRI scans) were plotted as shown in Figure 2. A “true” minimum (an average of all minimums/valleys, blue downward-facing triangles), mean (an average of all the temporal measures), and maximum (an average of all maximums/peaks, red upward facing triangles) dimensions of the airway were obtained at each NP or RG plane for every subject. These minimums, mean and maximums are the surrogate measures of temporal changes in airway in a patient and used for comparing the effects of dynamics changes in airway across patients.

### 2.4. Statistical Analysis

Patient demographics, hemodynamics, and outcome measures were summarized using descriptive statistics (mean and standard deviations or median and interquartile range for continuous variables and frequency and percentages for categorical variables). Univariate analysis was performed to compare the paired differences in mean, minimum, maximum outcome measures of the airway dimensions between the baseline (DEX alone) condition and at 5, 10, and 15 min after the addition of ketamine using paired *t*-tests or Wilcoxon signed-rank tests as appropriate. Linear mixed-effects models were also used to analyze the changes from their baseline values for each of the airway measures. The model specification was designed to test the difference in outcomes between the baseline (DEX alone); and, at 5, 10, 15 min after adding ketamine to the baseline anesthetic condition. The models included time (5, 10, 15 min), as well as the age of the study participant and the baseline airway measure as fixed effects to account for between-subject variations due to these factors. The models also included participant as a random effect to account for the dependency among observations of the same subject. Statistically significant differences were declared when *p* < 0.05.

### 2.5. Power Analysis

Power analysis was based on the two primary outcomes, cross-sectional area of the pharyngeal airway of the patients measured at soft palate (NP) and the base of tongue (RG) and estimates of differences and variability between the treatment groups was based on a previous study by Evans et al. [25]. An initial sample size of 28 and a final sample size of 22 (allowing 20% inadequate data due to low dose of anesthesia and/or movement of the patient during imaging) will have 80% power to detect a difference in the two primary outcomes with an effect size of 0.7 times the standard deviation of the change in outcomes. The power analysis was conducted using nQuery 6.0 using a two-sided paired *t*-test with a significant level of 0.025, adjusting for two comparisons on the two primary outcomes.

## 3. Results

Twenty-five children were recruited for this study but 3/25 were excluded because they failed the initial DEX sedation due to motion. Twenty-two children and young adults (82% male) ranged in age from 1 to 13 years with a median age of three (IQR = 2, 6) and had a median BMI of 17.4 kg·m${}^{-2}$ (IQR = 15.7, 17.7). All subjects had a UMSS score of either 2 or 3 after the dexmedetomidine loading dose.

### 3.1. Dynamic Changes in Airway Dimensions

**Figure 3:**
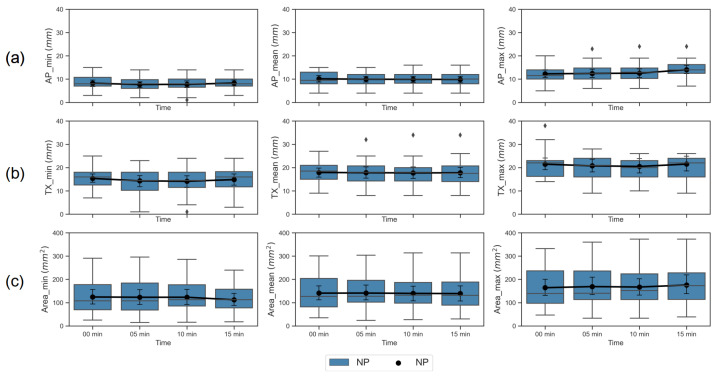
Box plot and a point plot with mean and 95% CI across 22 patients for each of the 9 airway dimensions (listed in Table 1) in nasopharyngeal (NP) plane. Airway measures at the four anesthetic conditions of 00 min (DEX only, baseline), 5, 10, and 15 min after administering ketamine at the baseline condition were plotted on the x-axis. Each of the subplots represent an airway measure, row-wise: (**a**) anterior-posterior (AP) diameter (in mm), (**b**) transverse (TX) diameter in (mm), and (**c**) cross-sectional area (in mm^2^) at the minimum (min), mean and maximum (max) airway sizes (plotted column-wise) during continuous breathing.

**Figure 4:**
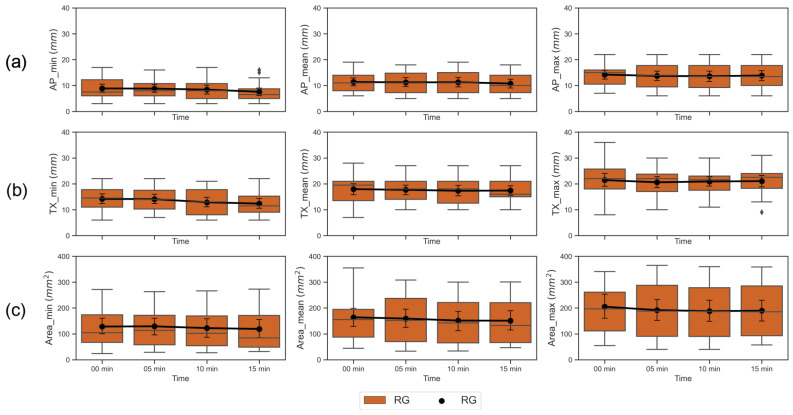
Box plot and a point plot with mean and 95% CI across 22 patients for each of the 9 airway dimensions (listed in Table 1) in retroglossal (RG) plane. Airway measures at the four anesthetic conditions of 00 min (DEX only, baseline), 5, 10, and 15 min after administering ketamine at the baseline condition were plotted on the x-axis. Each of the subplots represent an airway measure, row-wise: (**a**) anterior-posterior (AP) diameter (in mm), (**b**) transverse (TX) diameter in (mm), and (**c**) cross-sectional area (in mm^2^) at the minimum (min), mean and maximum (max) airway sizes (plotted column-wise) during continuous breathing.

The airway measures of cross-sectional area, transverse (TX) diameter, and anterior-posterior (AP) diameter at the four anesthetic conditions: DEX alone (00 min, baseline), and 5, 10, 15 min after ketamine administration to the baseline condition, at the level of the nasopharyngeal and retroglossal airways for 22 patients are shown as box plots in Figure 3 and Figure 4. The line plots in the Figure 3 and Figure 4 represent the changes in each airway measure, averaged across the 22 subjects in NP and RG airway planes respectively.

### 3.2. DEX Alone vs. DEX + Ketamine at 15 min: Paired T-test or Wilcoxon Signed Rank Test

The mean and standard deviations for each of the measures and treatment group is reported in Table 1. Table 1 also shows the mean and 95% confidence interval (CI) in differences of absolute measures and the percent changes in airway dimensions, 15 min after administering ketamine to the baseline condition of Dexmedetomidine alone. The differences between both the treatment groups are negligible, considering the variability in AP and TX diameters of 2 pixels (1 mm) and the cross-sectional area of 10% with the semi-automatic automatic airway segmentation approach. The differences between the treatment groups are also statistically not significant (p≥0.05) based on the Paired *T*-test for parametric data or Wilcox Signed Rank test for non-parametric data as appropriate. The effect sizes based on Cohen’s d is also small, except for AP diameter at maximum airway size in the nasopharynx, AP, and TX diameters at minimum airway size during a respiratory cycle at retroglossal. and small effect sizes in changes in airway dimensions suggest that there is probably no effect with the addition of ketamine to the DEX condition and the sample size is not an issue.

### 3.3. Comparison across Treatment Groups: Linear Mixed Effects Model

Subjects were tested for the differences in airway measures between baseline (DEX alone) condition and each of the 5, 10, and 15-min conditions after adding ketamine to the baseline condition. The linear mixed-effects model showed no evidence for overall time effect on the changes in airway dimensions from the baseline condition on all of the 24 measures (listed in Table 1), after adjustment for the age of the subject and the baseline airway measures. Least squares means at each time point showed that NP_Area_Maximum at 15 min (LSM (95% CI) = 18.6 (3.3, 34.0), *p* = 0.0184), NP_TX_Minimum at 10 min (LSM (95% CI) = −1.1 (−2.2, −0.1), *p* = 0.0401) and at 15 min (LSM (95%CI) = −1.2 (−2.3, −0.1), *p* = 0.0287), NP_AP_Maximum at 15 min (LSM (95%CI) = 1.4 (0.3, 2.6), *p* = 0.0181), NP_AP_Maximum at 15 min (LSM (95%CI) = 1.6 (0.6, 2.7), *p* = 0.0031) are significantly different from the baseline condition at *p* = 0.05. However, none of them is significant after multiplicity adjustment. This suggests that neither the addition of ketamine nor the length of time ketamine was administered in a patient seems to have any clinically significant effect on the airway dimensions, while the patient was initially anesthetized by Dexmedetomidine.

### 3.4. Hemodynamic Measurements

**Figure 5:**
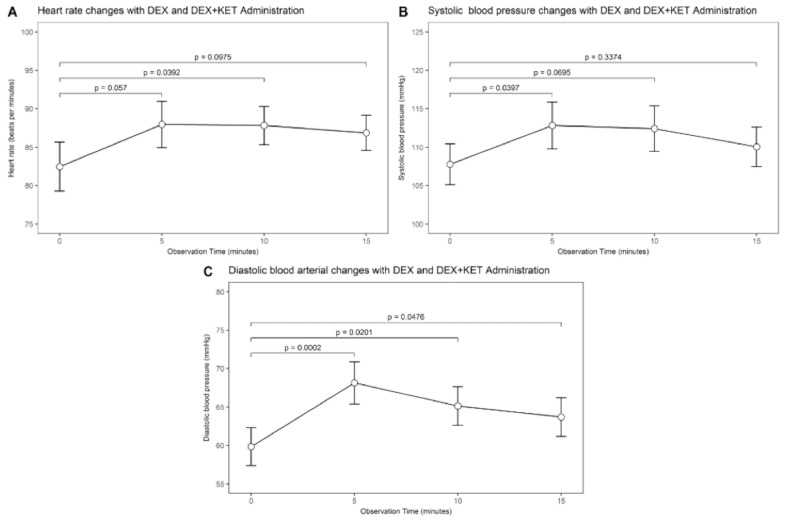
Changes hemodynamics with dexmedetomidine (baseline) and after ketamine administration. In the x-axis, the first data point is the mean baseline hemodynamic value, and the second, third, and fourth points are the mean hemodynamic value after 5, 10, and 15 min of ketamine administration. (**A**) Comparison of baseline and post ketamine heart rates. Compared with baseline, the administration of ketamine did increase the mean heart rate significantly only after 10 min of ketamine administration (*p* = 0.0392). (**B**) Comparison of baseline and post ketamine systolic blood pressures. Compared with baseline, the administration of ketamine did increase mean systolic blood pressure significantly after 5 min of ketamine administration (*p* = 0.0397) (**C**) Comparison of baseline and post ketamine diastolic blood pressures. Comparison of baseline and post ketamine diastolic blood pressures. Compared with baseline, the administration of ketamine did increase mean diastolic blood pressure significantly after 5 min of ketamine administration (*p* = 0.0002), 10 min (*p* = 0.0201), and 15 min (*p* = 0.0476)). All *p*-values were adjusted for multiple comparisons (DUNNETT adjustment for testing differences with a control level).

Mean arterial oxygen saturation remained unchanged with adding ketamine to dexmedetomidine. No subjects demonstrated clinical evidence of airway obstruction during the study or in the post-anesthesia care unit. Hemodynamic effects were observed with both DEX alone and after ketamine administration (Figure 5). Ketamine + dexmedetomidine administration increased mean heart rate (Figure 5a) significantly only after 10 min of administration (88 ± 12 bpm, *p* = 0.0392) compared with DEX alone (heart rate 82 ± 15 bpm). Ketamine + dexmedetomidine administration increased mean systolic blood pressure (Figure 5b) significantly after 5 min of administration (113 ± 14 mmHg, *p* = 0.0397) compared with DEX only (107 ± 12 mmHg). Ketamine + dexmedetomidine administration also increased mean diastolic blood pressure (Figure 5c) significantly after 5 min, (68 ± 13 mmHg, *p* = 0.0002), 10 min (65 ± 12 mmHg, *p* = 0.0201) and 15 min (64 ± 12 mmHg, *p* = 0.0476) compared with DEX only (59 ± 12 mmHg). All *p*-values were adjusted for multiple comparisons (DUNNETT adjustment for testing differences with a control level). None of the hemodynamic changes were considered to be of sufficient magnitude to necessitate interrupting or terminating the imaging study.

## 4. Discussion

The ability to maintain spontaneous ventilation and upper airway tone while maintaining stable hemodynamics makes the dexmedetomidine/ketamine combination an attractive choice for procedural sedation. Our results demonstrated statistically insignificant differences in upper airway dimensions both in nasopharyngeal and retroglossal axial planes 15 min after adding ketamine to dexmedetomidine anesthesia. Any dose-dependent decreases in the measures of airway caliber between baseline and 5 or 10 min after ketamine administration were small in both absolute and relative magnitude and were not associated with clinical evidence of airway obstruction.

Upper airway patency depends upon pharyngeal mechanics, neuromuscular activity, and luminal pressure [26]. Currently available sedative and anesthetic agents depress upper airway dilator muscle activity to varying degrees [27,28,29]. These agents induce unconsciousness by altering neurotransmission at multiple areas in the cerebral cortex, brain stem, and thalamus. The molecular targets for dexmedetomidine are the central alpha 2-adrenergic receptors. The sedative effects of dexmedetomidine are mediated via stimulation of the alpha 2A-receptor sub-type [30] in the locus ceruleus [31]. Studies of electroencephalogram activity during dexmedetomidine sedation in healthy adults and children report that dexmedetomidine sedation results in a state which closely resembles stage 2 sleep [32,33].

In a recent study, we compared airway collapsibility measured by the critical closing pressure obtained during natural sleep to that obtained under dexmedetomidine in patients with obstructive sleep apnea. This study concluded that airway collapsibility during normal sleep was not significantly different from collapsibility measured during sedation with dexmedetomidine. In the current prospective study, we excluded children with obstructive sleep apnea to avoid confounding effects of varying preexisting airway collapsibility of children with obstructive sleep apnea. Therefore, our results show the effects of dexmedetomidine and ketamine on upper airway morphology are generalizable only to children without obstructive sleep apnea [20]. However, our results may also apply to children with severe preoperative airway impairment since the combination of dexmedetomidine and ketamine has shown to be associated with a higher rate of successful completion and fewer oxygen desaturations during drug-induced sleep endoscopy, when compared to propofol, or sevoflurane plus propofol [12].

In the previous studies, we imaged the upper airway with MRI using 1 μg·kg${}^{-1}$·h of dexmedetomidine and a higher dose of 3 μg·kg${}^{-1}$·h. We found that airway dimensions were smaller at both doses. These changes in airway dimensions were small and were not associated with clinical airway obstruction [18]. In children with obstructive sleep apnea, increasing dexmedetomidine dosing was not associated with any significant changes in airway morphology [19]. Collectively, these studies reinforce the observation that dexmedetomidine provides relatively safe sedation to children.

Ketamine is an N-methyl-D-aspartate receptor antagonist causing dissociative anesthesia through actions within the limbic and thalamic systems. It has a rapid onset after IV/IM administration (less than 5 min), with a recovery time between 45 and 120 min. It is reported to have minimal effect on the central respiratory drive and is a bronchial smooth muscle relaxant [34]. A rat study found that electromyographic (EMG) genioglossus activity was increased with ketamine administration compared to activity during natural sleep and activity in rats treated with propofol [35]. The favorable effect of ketamine on airway collapsibility can be attributed to its pharmacologic sleep-inducing action. While γ-aminobutyric acid (GABA) ergic anesthetics (e.g., propofol) and benzodiazepines compromise the airway by muscle relaxation or respiratory drive suppression, ketamine does not [36]. In a recent randomized crossover trial, Mishima et al. [37] demonstrated that Ketamine causes increased neuromuscular activity and increased inspiratory duty cycle and hence, can maintain passive upper airway collapsibility and compensate for respiratory response against acute partial upper airway obstruction.

We studied children with clinically relevant doses of dexmedetomidine and ketamine during typical procedural sedation for noninvasive and minimally invasive procedures. Our data demonstrate a definite deep level of sedation as assessed by the UMSS. However, doses of both agents outside the dose range duration used in our study may have different effects.

One limitation of our study is that no baseline measurements were obtained in awake subjects. Although such baseline measurements would be ideal, we determined that attempting to obtain them would likely be impractical, inefficient, and ultimately unsuccessful. Like many, perhaps most children do not tolerate the MRI environment while awake, even under optimum conditions. Additionally, children are unlikely to lie completely still during the 2-min acquisition of the CINE MR images, especially given the extremely loud noises associated with the gradient-echo sequences. The second limitation of our study was that we examined the effect of adding an intravenous bolus of ketamine only for 15 min. However, A recent study examined the optimal sampling schedule for children receiving 1–1.5 mg·kg${}^{-1}$ intravenous bolus of ketamine for short-term procedural sedation and analgesia. They concluded that plasma concentrations of ketamine remained relatively constant after 16 min of ketamine administration [38]. The third limitation of this study is the lack of a control group sedated solely by the dexmedetomidine. However, we consider our measurements at 00 min with dexmedetomidine alone and before administering ketamine as the baseline measurements in our statistical analyses, which accounts for any between-subject bias. This study addressed only a single dose of ketamine during dexmedetomidine infusion. We did not examine the effect of adding ketamine infusion because ketamine infusion is not considered a standard of sedation/anesthesia care at our institution. It will be interesting as a future study to examine the effect of both agents on airway dimensions of children with airway impairment. With the automatic segmentation of the airway boundaries from the MR images using image processing algorithms, any intra-user and inter-user variability in the data is eliminated. Any sources of error with the airway boundary segmentation (about a pixel width perimeter) with the region-growing algorithm is consistent across the seventy-two frames of the CINE MRI scan at any anesthetic condition, and across patients. This would not have impacted our analysis.

## 5. Conclusions

Our study demonstrates that changes in upper airway caliber and hemodynamics, produced by adding ketamine to dexmedetomidine in children with normal airway are small in magnitude and are not associated with clinical signs of airway obstruction.

## Figures and Tables

**Table 1:** Airway diameters and cross-sectional area at dexmedetomidine (DEX alone, 00 min) and 5, 10, and 15 min after administering ketamine to the DEX alone (baseline) condition.

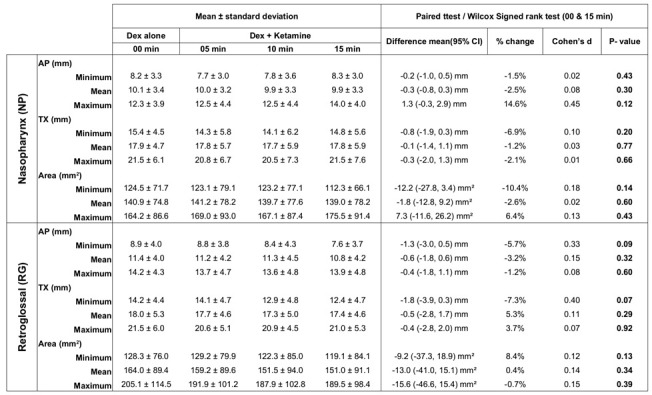

The airway measures in the axial planes of the nasopharyngeal and retroglossal airways are reported as a mean±standard deviation in 22 children. The changes in the airway dimensions between the baseline (DEX alone) and DEX + ketamine at 15 min were compared with the paired ttest or Wilcox signed-rank test as appropriate. The mean differences in airway measures along with 95% confidence interval (CI), percent change, and Cohens d effect sizes and *p*-values are reported. (NP: Nasopharynx, RG: Retroglossal, AP: Anterior-Posterior diameter, TX: Transverse diameter, statistically significance: *p* < 0.05).

## Abbreviations

The following abbreviations are used in this manuscript:

MRI	Magnetic Resonance Imaging
DEX	Dexmedetomidine
ASA	American Society of Anesthesiologists
IV	Intravenous
UMSS	University of Michigan Sedation Scale
NP	Nasopharynx
RG	Retroglossal
AP	Anterior-Posterior
TX	Transverse
BMI	Body Mass Index
IQR	Interquartile Range
CI	Confidence Interval
